# Enhanced recovery and abbreviated length of anticoagulation for thromboprophylaxis after primary hip arthroplasty rationale and design of the ENABLE-hip trial

**DOI:** 10.1007/s11239-025-03110-5

**Published:** 2025-05-29

**Authors:** Philipp Drees, Irene Schmidtmann, Manuel Herbst, Dorothea Becker, Stefano Barco, Frederikus A. Klok, Karsten Keller, Lukas Hobohm, Konstantinos C. Christodoulou, Christina Abele, Rupert Bauersachs, Walter Ageno, Erik Lerkevang Grove, Henrik Kehlet, Friedhelm Hufen, Thomas Klonschinski, Yama Afghanyar, Lukas Eckhard, Nadine Martin, Susanne Fischer, Stanislav Gorbulev, Dominik Rath, Anna C. Mavromanoli, Claude Jabbour, Irene Lang, Francis Couturaud, Christian Heiss, Harald Binder, Stavros Konstantinides

**Affiliations:** 1 Center for Orthopedics and Trauma Surgery, University Medical Center of the Johannes Gutenberg University, Mainz, Germany; 2 Institute of Medical Biostatistics, Epidemiology and Informatics, University Medical Center of the Johannes Gutenberg University, Mainz, Germany; 3 Center for Thrombosis and Hemostasis, University Medical Center of the Johannes Gutenberg University, Mainz, Germany; 4 Department of Angiology, University Hospital Zurich, Zurich, Switzerland; 5 Department of Medicine—Thrombosis and Hemostasis, Leiden University Medical Center, Leiden, The Netherlands; 6 Department of Cardiology, University Medical Center of the Johannes Gutenberg University, Mainz, Germany; 7 Cardioangiologic Center Bethanien CCB, Frankfurt Am Main, Germany; 8 Department of Internal Medicine, University of Padua, Padua, Italy; 9 Department of Cardiology, Aarhus University Hospital, Aarhus, Denmark; 10 Department of Clinical Medicine, Faculty of Healthy, Aarhus University, Aarhus, Denmark; 11 Section for Surgical Pathophysiology, Rigshospitalet, Copenhagen, Denmark; 12 Mainz, Germany; 13 Joint Surgery Center, Bad Kreuznach, Germany; 14 Interdisciplinary Center for Clinical Trials, University Medical Center of the Johannes Gutenberg University, Mainz, Germany; 15 Department of Cardiology and Angiology, University Hospital Tübingen, Tübingen, Germany; 16 University Hospital Zurich, Zurich, Switzerland; 17 Department of Cardiology, Angiology, Haemostaseology and Medical Intensive Care, First Department of Medicine, Medical Faculty Mannheim, University Medical Centre Mannheim, Heidelberg University, Mannheim, Germany; 18 Department of Internal Medicine II, Medical University of Vienna, Cardiology, Austria; 19 Univ_Brest, INSERM U1304-GERBI, CIC INSERM, 1412 Brest, France; 20 Département de Médecine Interne Et Pneumologie, CHU Brest, Brest, France; 21 FCRIN INNOVTE Network, Saint-Etienne, France; 22 Trauma, Hand and Reconstructive Surgery, Justus Liebig University, Giessen, Germany; 23 Institute of Medical Biometry and Statistics, Faculty of Medicine and Medical Center, University of Freiburg, Freiburg, Germany

**Keywords:** Venous thromboembolism, Randomized controlled trial, Total hip arthroplasty, Thromboprophylaxis, Anticoagulation

## Abstract

Surgical total hip arthroplasty (THA) is associated with high risk of venous thromboembolism (VTE), but the appropriate duration of postoperative anticoagulation remains controversial. “Enhanced Recovery and Abbreviated Length of Anticoagulation for Thromboprophylaxis After Primary Hip Arthroplasty” (ENABLE-Hip) is a multicenter investigator-initiated and academically sponsored randomized double-blind active-control non-inferiority trial. Patients will be mobilized early after surgery, following a standardized enhanced recovery protocol. After an initial open-label prophylactic anticoagulation as per local standard of care until day 2 after surgery, treatment with rivaroxaban (10 mg once daily) will be started on day 3 and continued until day 10. Subsequently, patients will be switched to placebo in the experimental arm, or continue on active drug in the control arm, until a total of 35 days. The primary endpoint is acute symptomatic or fatal VTE within 3 months. A sample size of 2,932 patients will provide ≥ 80% power to reject the null hypothesis that δ ≥ 0.01 (δ = difference between the two arms in symptomatic VTE probability) at a significance level α = 0.05. An interim analysis will be performed after 3-month follow-up of the first 1,760 randomized patients at a significance level α = 0.50, leading to stop for futility if significance is not obtained, or if recalculation yields a sample size of > 3,200 patients. ENABLE-Hip will be the first major randomized trial to test an overall reduction in the duration of post-THA thromboprophylaxis and will inform future guideline recommendations concerning this continuously growing patient population.

**Trial registration:** ClinicalTrials.gov Identifier: NCT06611319

**Graphical Abstract:**

Flow diagram of the ENABLE-Hip trial (ClinicalTrials.gov Identifier: NCT06611319).

ERAS: enhanced recovery after surgery; *o.d*.: *omni die* (once daily).

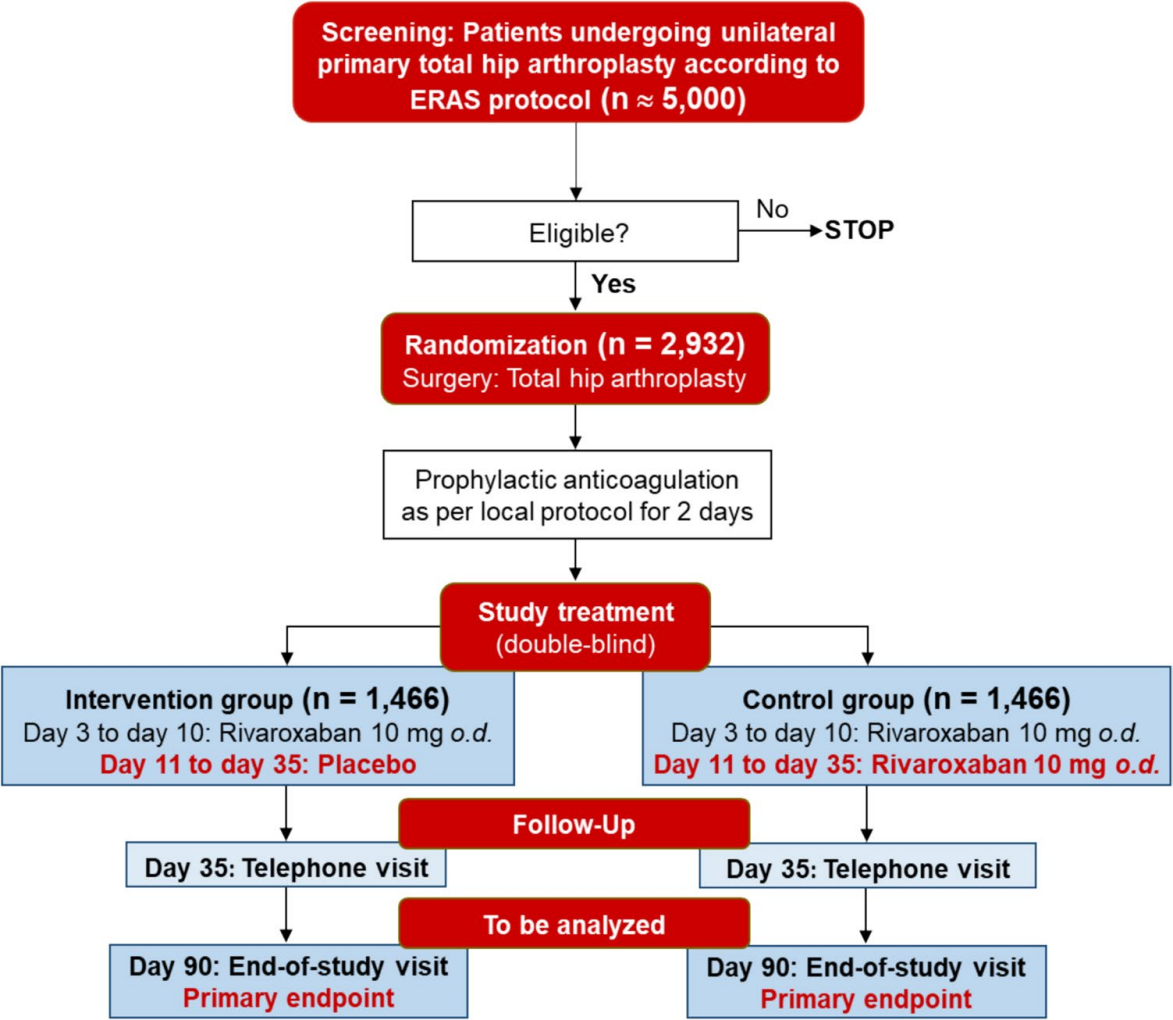

## Highlights

- The number of patients requiring a surgical joint arthroplasty is constantly increasing.
- ENABLE-Hip is a rage multicenter multinational randomized trial testing the hypothesis that a shorter duration of anticoagulant treatment is non-inferior to the current standard of care in preventing symptomatic or fatal venous thromboembolism in patients after hip arthroplasty.
- The trial will inform future national and international guidelines for this large and continuously growing patient population, and may improve patient care by reducing the need for anticoagulation and its associated complications.

## Introduction

An increasing proportion of the ageing population in Europe and other parts of the world suffers from osteoarthritis and will need joint arthroplasty at some time in their lives. In the United States, more than one million total joint arthroplasty procedures are performed each year [1]. In Europe, an analysis of the German nationwide inpatient statistics revealed that the annual number of patients undergoing total hip arthroplasty (THA) increased from 145,223 in the year 2005 to 171,421 in 2016 [2]. THA is a surgical procedure with a high risk of venous thromboembolism (VTE). Early data reported postoperative VTE rates, including asymptomatic deep vein thrombosis (DVT), as high as 84% without thromboprophylaxis [3]. As a result, experts and guidelines recommend pharmacologic VTE prophylaxis as the standard of care [4–8]. However, the VTE risk and consequently the appropriate duration of anticoagulation may vary, depending on advances in surgical techniques, the speed of postoperative mobilization, and the length of hospital stay. In this context, improved surgical techniques and early recovery protocols (so-called *fast-track* surgery) may help to reduce the incidence of postoperative VTE [9, 10]. To achieve this goal, enhanced recovery programs have been developed and are increasingly implemented in the surgical treatment of knee and hip osteoarthritis. An interdisciplinary team of orthopaedic surgeons, anesthesiologists, nurses and physiotherapists involves the patient as an active member of the perioperative management with the aim of promoting early postoperative mobility and reducing morbidity and the length of hospital stay [11].

The appropriate duration of anticoagulation following elective THA is a controversial, dynamically evolving topic. Country-specific recommendations and physician as well as patient preferences have a major impact on postoperative management with regard to thromboprohylaxis. For example, in Germany, interdisciplinary guidelines last updated in 2015 [12], advocate the administration of a low-molecular weight heparin (LMWH), fondaparinux, or an approved direct oral anticoagulant (DOAC) at a prophylactic dose for 28–35 days after THA. In contrast, in North America, both older [6, 7] and updated recommendations encourage acetylsalicylic acid (ASA) prescriptions by orthopedic specialists [8].

Over the last ten years, a number of studies compared various thromboprophylaxis regimens in patients after a hip or knee arthroplasty. Two of them need particular mentioning due to their design and their size:

A large multicenter double-blind randomized controlled trial (RCT) involving 3,424 patients (1,804 undergoing THA and 1,620 undergoing total knee arthroplasty, TKA) was conducted in Canada. Among patients who received 5 days of postoperative rivaroxaban, extended prophylaxis with ASA was not significantly different from continued rivaroxaban in the prevention of symptomatic VTE; primary event rates were low (≤ 0.70%) in both treatment arms [13]. Of note, the mean length of hospital stay was short (< 4 days) in that trial; this is still in contrast to clinical practice in some European countries, in which postoperative stay is generally longer [2], approaching 4 days only in selected centers [11]. Moreover, although major bleeding complications were infrequent in both groups, they were numerically more frequent (8 versus 5) in the ASA compared to the standard anticoagulation group [13], generating questions on whether there is any clinically relevant benefit by replacing an anticoagulant with ASA.

More recently, a cluster-randomized, crossover, registry-nested trial across 31 hospitals in Australia examined whether ASA was non-inferior to enoxaparin in preventing symptomatic VTE after THA or TKA [14]. Enrollment was stopped after an interim analysis determined the stopping rule was met, with 9,711 of the prespecified 15,562 patients enrolled (62%); of those, 9,203 (95%) completed the trial. Within 90 days of surgery, the symptomatic VTE rate in the ASA group was 3.45% compared to 1.82% in the enoxaparin group (estimated difference, 1.97%; 95% CI, 0.54%-3.41%). This failed to meet the non-inferiority criterion for ASA, being significantly superior for enoxaparin (P = 0.007) [14]. Currently, the ongoing Extended Venous Thromboembolism Prophylaxis Comparing Rivaroxaban and Aspirin to Aspirin Alone Following Total Hip and Knee Arthroplasty (EPCAT) III trial (NCT04075240) is comparing 35 days of ASA (without an anticoagulant) with 5 days of rivaroxaban followed by 30 days of ASA in patients undergoing THA.

In view of the existing data, it has been proposed to limit anticoagulation to the in-hospital period after *fast-track* THA and discontinue it upon discharge without replacement by an antiplatelet agent; in fact, the positive results of cohort studies performed in Denmark [10, 15] led to an endorsement of short, in-hospital-only thromboprophylaxis in that country [16]. Nevertheless, broader acceptance of this approach necessitates robust, large-scale controlled data [17–21].

### Study design and objectives

ENABLE-Hip (ClinicalTrials.gov Identifier: NCT06611319) is an academic prospective randomized double-blind active-control non-inferiority trial. Sponsor is the University Medical Center of the Johannes Gutenberg University, Mainz, Germany. The trial tests the hypothesis that a *shorter duration* of anticoagulant treatment is non-inferior, in terms of preventing symptomatic or fatal VTE, compared to the current standard of care. Specifically, a regimen of short (over 10 days) prophylactic anticoagulation, postulated to be effective and safe after fast-track THA on the basis of existing data as mentioned above, will be compared to standard-duration (35 days) anticoagulation as per current guidelines. The double-blind design will minimize the risk of unjustified diagnostic work-up (particularly for DVT) in patients without new-onset symptoms in the experimental arm, and participating investigators will be advised to adhere to guideline recommendations in this regard [22, 23].

### Patient population and eligibility

Adult patients, aged 18 to 85 years, undergoing elective unilateral primary THA, who fulfill the criteria for enhanced (fast-track) recovery based on a predefined multidisciplinary plan of care will be included in the ENABLE-Hip trial [11]. The key inclusion and exclusion criteria are listed in Table 1.

**Table 1:** Key inclusion and exclusion criteria

Inclusion criteria	Exclusion criteria
1) Written informed consent 2) Age between 18 and 85 years 3) Scheduled to undergo elective unilateral primary THA and eligible for perioperative management based on the ERAS protocol 4) Baseline TUG test scoring < 20 s, corresponding to a good mobility status before surgery 5) Capability to understand and comply with the protocol requirements (e.g., to answer the questionnaires) 6) Negative serum pregnancy test and and highly effective method of contraception for the duration of study treatment	1) Previous DVT or PE 2) Hip or lower limb fracture in the previous three months 3) Major surgical procedure within the previous three months 4) Active cancer (metastatic or requiring chemotherapy or radiation therapy) 5) Active peptic ulcer disease, gastritis, or prior gastrointestinal bleeding 6) Obesity with body mass index (BMI) > 40 kg/m^2^ body surface area 7) Severe renal impairment defined as estimated glomerular filtration rate < 30 ml/min 8) Severe hepatic impairment defined as Child Pugh Class B or C 9) Uncontrolled intercurrent illness (i.e., active infection, symptomatic congestive heart failure, uncontrolled hypertension, unstable angina pectoris, interstitial lung disease, serious gastrointestinal conditions, psychiatric illness) 10) Active or recent major bleeding at any site, or any disease or condition which, in the investigator’s jedgement, may significantly increase the bleeding risk during postoperative anticoagulation 11) Contraindication to discharge within 6 days after surgery 12) Expected requirement for major surgery within a 90-day period post THA 13) Need for long-term anticoagulation (e.g., atrial fibrillation, previous VTE) 14) Need for chronic antiplatelet therapy except for acetyl salicylic acid at a dose ≤ 100 mg daily or clopidogrel 75 mg daily 15) Previous participation in this trial 16) Life expectancy < 6 months 17) Participation in another interventional clinical trial at inclusion or within the last 30 days prior to inclusion, except during the observational follow-up period of that rial 18) History of hypersensitivity to the investigational medicinal product or to any drug with similar chemical structure or to any excipient in its pharmaceutical form

DVT: deep vein thrombosis; ERAS: enhanced recovery after surgery; PE: pulmonary embolism; THA: total hip arthroplasty; TUG: Timed Up and Go; VTE: venous thromboembolism

Informed consent by the participants will be necessary for documentation and evaluation of their data. Subjects can withdraw their consent at any time during the trial and without giving reasons, and this will not entail any disadvantages for them. If possible, subjects having withdrawn consent will be followed, and all examinations scheduled for the end-of-study visit will be performed and documented for inclusion in the statistical analysis.

### Intervention and treatment regimens, trial flow

In the experimental intervention arm, patients will be mobilized early after THA surgery, following the standardized Early Recovery After Surgery (ERAS) protocol [11]. Following a brief initial open-label period of prophylactic anticoagulation (to allow for subcutaneous low molecular weight heparin if mandated by local treatment protocols) until day 2 after surgery, treatment with the study drug (rivaroxaban at the standard, approved prophylactic oral dose of 10 mg once daily) will be started on the third postoperative day and continued for 8 days (i.e., until day 10 after surgery). After this time, patients will be switched (in a double-blinded manner) to placebo, to be continued for 25 days (until day 35 after surgery).

In the control arm, patients will follow the same standardized enhanced recovery protocol as the experimental group. Following a brief initial open-label period of prophylactic anticoagulation as per local standard of care (to allow for subcutaneous low molecular weight heparin if mandated by local treatment protocols) until day 2 after surgery, treatment with the study drug (rivaroxaban at the standard, approved prophylactic oral dose of 10 mg once daily) will be started on the third postoperative day and continued for 8 days (i.e., until day 10 after surgery). After this time, patients will continue (in a double-blinded manner) to receive rivaroxaban at the above mentioned dose for 25 days (i.e., until day 35 after surgery).

The trial flow diagram is shown in Graphical Abstract. After confirmation of eligibility criteria (Table 1) and obtaining written informed consent, subjects will be randomly assigned to one of the above treatment groups. Treatment assignment will be performed via the electronic case report form (eCRF) on Visit 1 (day -10 to day -1 before surgery). A randomization/treatment assignment list will be generated by the Institute of Medical Biostatistics, Epidemiology and Informatics (IMBEI) at the University Medical Center Mainz. The randomization ratio of the experimental treatment group and the control treatment group will be 1:1. In order to account for possible differences in the initial open-label period of prophylactic anticoagulation as per local standard, randomization will be performed per site. Additionally, randomization will be stratified by concomitant single chronic antiplatelet medication (ASA or clopidogrel). Block randomization with varying block length will be applied. Considering that 10–15 centers are expected to recruit a total of 2,932 patients into the trial (see below), we anticipate 20 strata with sizes between 100 and 150.

### Outcomes

The trial visit plan is displayed in Table 2; the primary and secondary endpoints are described in Table 3. The primary efficacy endpoint is acute symptomatic or fatal VTE, defined as (i) symptomatic DVT of the popliteal or more proximal leg veins (femoral or iliac veins) or inferior vena cava; or (ii) symptomatic (segmental or more proximal) or fatal PE, occurring within the first 90 days after surgery and confirmed by objective testing. Physicians will be advised to use standardized clinical prediction rules and diagnostic algorithms for *clinically suspected* VTE as recommended by current guidelines [22, 23]. Patients with suspected DVT will undergo compression sonography from the common femoral vein to the trifurcation of the popliteal vein. Patients with suspected PE will undergo computed tomography pulmonary angiography or ventilation-perfusion lung scan or invasive pulmonary angiography. No screening or diagnostic tests will be performed in asymptomatic patients.

**Table 2:** Trial visit plan and data collection schedule

Visits	Visit 1 (Screening and Enrolment)	Visit 2 (Surgery)	Visit 3 (Discharge)	Visit 4 (EOT)	Visit 5 (EOS)
Action					
Trial day	Day-10 to -1	Day 0	Day 2–6	Day 35 (+ 7 days)	Day 90 (± 10 days)
Type of visit	On site	In hospital		Telephone contact	On site
Demographics (e.g., sex, age)	X				
Subject information and written informed consent	X				
Medical history	X				
Inclusion/exclusion criteria	X				
Physical examination^a^	X		X		X
Vital signs^b^	X		X		
Clinical chemistry^c^	X				
Hematology^d^	X				
Serum pregnancy test^e^	X				
Dispense of urine pregnancy test^e^			X		
Urine pregnancy test^e^				X	
Range of motion (active)	X		X		X
Timed Up and Go (TUG) score	X		X		X
Allocation of study medication and patient number via eCRF	X				
Patient questionnaires					
• Hip disability and Osteoarthritis Outcome Score (HOOS-12)	X				X
• EQ-5D-5L (generic quality of life)	X				X
• Health care resources utilization				X	
Dispense of study medication^f^		X	X		
Observation and documentation of					
• Symptomatic/fatal venous thromboembolism^g^		X	X	X	X
• Major and clinically relevant non-major bleeding		X	X	X	X
• Stroke (haemorrhagic/ischemic)		X	X	X	X
• Other Adverse Events (AEs)	X	X	X	X	X
• Concomitant medication/therapies	X	X	X	X	X
End of trial (final visit)					X

EOS: end of study; EOT: end of treatment

^a^Examination regarding signs of orthopnea, moist rales/pulmonary edema, distended neck veins and leg vein thrombosis

^b^Blood pressure, pulse rate, respiratory rate, arterial or capillary blood oxygen saturation, body temperature

^c^Sodium, potassium, calcium, magnesium, chloride, glucose, creatinine, estimated glomerular filtration rate (eGFR), alanine aminotransferase (ALT), γ-glutamyltransferase (γ-GT), international normalized ratio (INR), activated partial thromboplastin time (aPTT)

^d^Leukocyte (total white blood cell count, basophils, eosinophils, neutrophils, monocytes, lymphocytes), erythrocyte and platelet count, hemoglobin, hematocrit

^e^Required for women of childbearing potential (i.e., all women until 2 years post-menopausal)

^f^The study medication kit will be dispensed once, either at Visit 2 or Visit 3, but in any case, before or on day 3 after surgery

^g^Diagnostic work-up of clinically suspected deep vein thrombosis or pulmonary embolism as per current guideline recommendations (no screening in asymptomatic patients)

**Table 3:** Primary and secondary endpoints

Primary endpoint	Secondary endpoints
Acute symptomatic or fatal VTE, defined as (i) symptomatic DVT of the popliteal or more proximal leg veins (femoral or iliac veins) or inferior vena cava; or (ii) symptomatic (segmental or more proximal) or fatal PE, occurring in the first 90 days after surgery and confirmed by objective testing Safety endpoint: Overt major or clinically relevant non-major bleeding	(1) Death from any cause (2) Isolated symptomatic distal DVT (3) Myocardial infarction or stroke (4) Need for readmission to the hospital (5) Length of hospital stay (6) Serious adverse events (SAEs) (7) Patient mobility (8) Changes in patient-reported hip joint-specific disability measured by the HOOS-12 score following surgery (9) Generic quality of life measured by EQ-5D-5L All within 3 months of the surgical intervention and using standardized scores and questionnaires Postoperative healthcare resource utilization will be assessed at 35 days after surgery

DVT: deep vein thrombosis; ERAS: Enhanced Recovery After Surgery (protocol); HOOS: Hip disability and Osteoarthritis Outcome; PE: pulmonary embolism

The safety endpoint is overt major or clinically relevant non-major bleeding. An overt bleed will be categorized as major if it fulfils at least one of the following criteria [24, 25]: (i) fatal; (ii) bleeding into a critical area or organ; (iii) surgical site bleeding that causes hemodynamic instability; (iv) non-surgical site bleeding causing a fall in hemoglobin level of ≥ 20 g L^−1^, or leading to transfusion of ≥ 2 units of whole blood or red blood cells; (v) surgical site bleeding that requires a second intervention (open, arthroscopic, endovascular), or hemarthrosis of sufficient size to delay mobilization or wound healing. Bleeding will be categorized as clinically relevant non-major if it does not meet the criteria for major bleeding, but results in hospitalization, aspiration of a wound, or a wound hematoma complicated by infection or any other non-major bleeding requiring medical attention. All other overt bleeding events will be considered minor.

Both the primary efficacy endpoint and the safety endpoint will be adjudicated by an independent critical events committee (CEC).

The EQ-5D-5L is a patient-reported outcome measure that provides a simple descriptive profile and a single index value for health status. The questionnaire entails 5 dimensions, including mobility, self-care, usual activities, pain/discomfort and anxiety/depression [26]. Patients will be asked to complete the questionnaire at baseline (Visit 1) and 90 days after surgery (Visit 5) (Table 2). The questionnaire will be administered at the beginning of the trial Visit, prior to any interaction with the trial investigator, including any tests, treatments or receipt of test/examination results. This is to avoid any possible influence on the subjects or their responses to the quality-of-life (QoL) questionnaire.

The Hip disability and Osteoarthritis Outcome Score (HOOS)-12, developed as a 12-item short form of the original 42-item HOOS [27], is one of the most widely used patient-reported outcome measures surrounding total hip arthroplasty [28, 29]. It assesses pain, function and quality of life. Patients will be asked to complete the questionnaire at baseline (Visit 1) and 90 days after surgery (Visit 5) (Table 2).

Utilization of health care resources will be recorded up to 35 days after surgery. Key parameters of health care resources utilization will be evaluated from a payers’ perspective and comprise visits to health care providers (general practitioner, medical specialists, etc.), rehospitalization as well as employment status and, if applicable, duration of inability to work (up to/more than 6 weeks) [30]. At Visit 3 (discharge), study participants will be asked to record all physician contacts/visits and medical treatments or healthcare services they received following discharge from the study site, and to provide relevant information at Visit 4 (at 35 days after surgery; Table 2).

### Sample size calculation and statistical analysis

The ENABLE-Hip trial has been designed as a two-arm non-inferiority trial for a binary study endpoint with a single interim analysis. The required total sample size needed to ensure a global one-sided significance level of 5% and a global power of 80% was computed using a normal approximation. The adaption strategy is based on a group sequential design with an interim analysis when 60% of the initially planned patients have completed the 3-month follow-up. Sample size recalculation using the conditional error function approach is planned at the interim analysis, and stop for futility is possible. No early stop for efficacy is planned.

A sample size of 2,786 evaluable patients has been calculated. Taking into account a possible censoring (i.e., dropout) rate of 5%, 2,932 patients need to be included in the trial. The assumptions for calculating the sample size were based on largely consistent existing data. More specifically, a pooled analysis[31] of 14 randomized controlled trials cited by the American College of Clinical Pharmacy (ACCP) guidelines on VTE prophylaxis after orthopedic surgery [5], yielded a symptomatic DVT rate of 1.27% after prophylactic LMWH use. Data from a prospective database of hospital-acquired VTE after THA or TKA in the United Kingdom revealed a similar VTE (DVT or PE) rate of 1.11% [32]. Based on these data and on Canadian experience, a large randomized controlled trial on brief (5-day) postoperative anticoagulation followed by ASA versus standard-duration anticoagulation assumed a 3-month VTE probability of 1.0% in the standard-of-care arm [13]. Although that trial eventually found VTE in only 0.64–0.70% of the patients in either arm [13], a 1.0% probability in the control arm remains an appropriate estimate as the results of the other studies mentioned above appear to be supported by a registry on total joint arthroplasty in the United States [33]. For the experimental arm, the estimated rate of ≥ 2% as per **H**_**0**_ corresponds to a ‘minimal clinically significant difference’ of 1.0% compared to the standard of care. This margin is medically sensible, considering that approximately 1 out of 25 (post-THA) VTE events that *might* occur due to a shorter anticoagulation period may be life-threatening, which has to be viewed against an up to 10% expected case-fatality rate of major hemorrhage associated with (longer) anticoagulation (reviewed in [13]). Finally, the assumption of a low frequency (1.0% or even less) of VTE events as per H_1_ is justified by the results of a nationwide prospective Danish cohort study, in which anticoagulation treatment (not followed by ASA) was given for the duration of hospitalization if it was less than 5 days [15].

The primary efficacy endpoint will be analyzed in the per-protocol (PP) population and in the modified intention-to-treat population (mITT). The PP population consists of all patients who have (i) signed the informed consent; (ii) undergone randomization and the planned surgical procedure (THA); and (iii) completed the study without major protocol deviations. The mITT population includes all randomized patients who underwent THA and received at least one dose of the study medication.

In case of poor protocol adherence, the duration of study treatment would be shortened similarly in both treatment arms (since this is a double-blind trial), and this would affect efficacy particularly in the control intervention arm in which active treatment duration is longer. On the other hand, if there were protocol violations leading to arbitrary discontinuation of study treatment and prescription of open-label rivaroxaban after day 10, these would happen with equal frequency in both groups, again given the double-blind design. Either scenario would result in attenuation or elimination of the differences in the ITT population, favoring demonstration of ‘non-inferiority’. For these reasons, testing for non-inferiority in the PP population is considered the most rigorous approach in such a setting [34, 35]. Nonetheless, as a European Medicines Agency (EMA) Reflection Paper suggests that the full analysis set (ITT) and the PP analysis set both have equal importance [36], the primary endpoint will be analyzed in both populations and non-inferiority will be declared if it can be established in both.

H_0_: p_E_–p_C_ ≥ δ, with δ = 0.01, where p_E_ and p_C_ are symptomatic VTE probability within 3 months in the experimental (p_E_) and control group (p_C_) respectively, will be tested against H_1_, p_E_–p_C_ < δ, with δ = 0.01. The test will be implemented by comparing the cumulative incidence of VTE at 3 months using the complementary log–log transformation [37]. Comparisons will be adjusted for stratum. Point estimates of the cumulative incidence will be obtained using the Kaplan–Meier method. To account for patients completing only part of the follow-up, tests based on the cumulative incidence will be used.

Categorical outcomes will be described by absolute and relative frequencies in each treatment group with the corresponding 95% confidence interval (CI). Comparisons will be made using logistic regression with treatment as explanatory variable and adjusting for stratum. Quantitative outcomes will be summarized by treatment group as means with standard deviation, and/or as medians with minimum, maximum values and interquartile range, as appropriate. Comparisons will be based on linear regression models with treatment as explanatory variable and adjusting for stratum, or on stratified Wilcoxon-Mann–Whitney tests. Time-to-event outcomes will be described by Kaplan–Meier estimates in each treatment group and compared using stratified log-rank tests. Exploratory tests will be two-sided at the 5% significance level and the CI will be two-sided 95% CI unless stated otherwise.

### Interim analysis

After 3-month follow-up of the first 1,760 randomized patients, a formal interim analysis will be performed on the PP population by an independent statistician at a significance level α = 0.50, leading to stopping for futility if significance is not obtained. Based on the results of the interim analysis, the conditional power (CP) of the final analysis will be calculated. If CP > 90%, sample size will be reduced to provide 90% CP. The trial will also be stopped for futility if CP ≤ 50% or recalculation at interim analysis yields an overall sample size of > 3,200 patients (including follow-up losses) to obtain 80% CP. The first condition for stop for futility corresponds to a promising zone with a lower bound of CP_min_ = 50% which guarantees that the significance level is kept when the conventional test statistic is used [38]. The second condition is due to feasibility of recruitment. The sample size re-calculation will be performed according to [38].

## Implications and expected impact

ENABLE-Hip is the first major multicenter randomized controlled trial to directly test an *overall reduction* in the duration of post-THA prophylactic anticoagulation instead of simply replacing one antithrombotic drug or regimen by another. No other RCT is currently testing a similar change in thrombosis-preventing strategy. In the United States, the ‘Comparative Effectiveness of Pulmonary Embolism Prevention After Hip and Knee Replacement (PEPPER)’ study (Clinicaltrials.gov: NCT02810704) is still ongoing, but unlike the present study, its aim is to compare different anticoagulation drugs and regimens, not different thromboprophylaxis periods. Thus, ENABLE-Hip has the potential to inform future national and international guidelines for this large and continuously growing patient population. If the trial yields positive results, i.e. demonstrates non-inferiority of the experimental intervention (10 days of prophylactic anticoagulation) compared to the current standard of care (anticoagulation over 28–35 days), reduction of anticoagulation duration by more than three weeks may, in view of the size of the patient population affected, save patients and the healthcare system a large number of days on unnecessary and potentially harmful (due to associated bleeding risks) anticoagulation each year.

## Current enrollment status

As of March 2025, six sites have been initiated, and a total of 59 patients have been enrolled. Currently estimated completion of enrollment is June 2027.

## Steering committee

Philipp Drees, Mainz, Germany (Co-Chair); Stavros Konstantinides, Mainz, Germany (Co-Chair); Stefano Barco, Zurich, Switzerland; Dorothea Becker, Mainz, Germany; Karsten Keller, Mainz, Germany; Frederikus A. Klok, Leiden, The Netherlands; Rupert Bauersachs, Frankfurt, Germany; Walter Ageno, Padua, Italy; Erik Lerkevang Grove, Aarhus, Denmark; Lukas Hobohm, Mainz, Germany; Henrik Kehlet, Copenhagen, Denmark; Christina Abele, Mainz, Germany; Friedhelm Hufen, Mainz, Germany.

## Trial statisticians

Irene Schmidtmann, Mainz, Germany; Christina Abele, Mainz, Germany; Philipp Mildenberger, Mainz, Germany.

## Data safety monitoring board (DSMB)

Irene Lang, Vienna, Austria (Chairperson); Francis Couturaud, Brest, France; Christian Heiss, Giessen, Germany; Harald Binder, Freiburg, Germany (DSMB Statistician).

## Critical events committee (CEC)

Dominik Rath, Tuebingen, Germany; Anna Mavromanoli, Zurich, Switzerland; Claude Jabbour, Mannheim, Germany.

## Data Availability

No datasets were generated or analysed during the current study.
